# Transcriptome Profiling and Toxicity Following Long-Term, Low Dose Exposure of Human Lung Cells to Ni and NiO Nanoparticles—Comparison with NiCl_2_

**DOI:** 10.3390/nano10040649

**Published:** 2020-03-31

**Authors:** Anda R. Gliga, Sebastiano Di Bucchianico, Emma Åkerlund, Hanna L. Karlsson

**Affiliations:** 1 Institute of Environmental Medicine, Karolinska Institutet, 171 77 Stockholm, Sweden; dibucchianico@helmholtz-muenchen.de (S.D.B.); emma.akerlund@ki.se (E.Å.); hanna.l.karlsson@ki.se (H.L.K.); 2 Comprehensive Molecular Analytics, Helmholtz Zentrum München, 81379 Munich, Germany; 3 Department of Oncology and Pathology, Science for Life Laboratories, Karolinska Institutet, 171 77 Stockholm, Sweden

**Keywords:** nickel nanoparticles, genotoxicity, long-term exposure, RNA sequencing

## Abstract

Production of nickel (Ni) and nickel oxide (NiO) nanoparticles (NPs) leads to a risk of exposure and subsequent health effects. Understanding the toxicological effects and underlying mechanisms using relevant in vitro methods is, therefore, needed. The aim of this study is to explore changes in gene expression using RNA sequencing following long term (six weeks) low dose (0.5 µg Ni/mL) exposure of human lung cells (BEAS-2B) to Ni and NiO NPs as well as soluble NiCl_2_. Genotoxicity and cell transformation as well as cellular dose of Ni are also analyzed. Exposure to NiCl_2_ resulted in the largest number of differentially expressed genes (197), despite limited uptake, suggesting a major role of extracellular receptors and downstream signaling. Gene expression changes for all Ni exposures included genes coding for calcium-binding proteins (*S100A14* and *S100A2*) as well as *TIMP3*, *CCND2*, *EPCAM*, *IL4R* and *DDIT4*. Several top enriched pathways for NiCl_2_ were defined by upregulation of, e.g., interleukin-1A and -1B, as well as Vascular Endothelial Growth Factor A (*VEGFA*). All Ni exposures caused DNA strand breaks (comet assay), whereas no induction of micronuclei was observed. Taken together, this study provides an insight into Ni-induced toxicity and mechanisms occurring at lower and more realistic exposure levels.

## 1. Introduction

Today, companies are producing nickel (Ni) and nickel oxide (NiO) nanoparticles (NPs), leading to a risk of exposure and possibly also to subsequent health effects [1,2]. From the historical industrial exposures to dust containing Ni, it is known that such exposure can cause various health effects, including cancer [3]. An increased risk of lung tumors has been observed in epidemiological studies both for soluble and insoluble Ni particles, but in studies on rats, only insoluble compounds (NiO, crystalline nickel subsulfide) demonstrated the induction of lung tumors in exposed rats [4]. This has been explained by a rapid clearance of soluble nickel and limited cell uptake, further proposing that the nickel species with the largest potential to induce tumors are those that (i) are insoluble enough to be present in the lung as particles, (ii) efficiently can enter epithelial cells via phagocytosis, and (iii) release Ni^2+^ ion in the phagosomes that, subsequently, can interact with DNA and proteins in the nucleus [4,5], also known as the Ni bioavailability model. The different physicochemical properties and biological effects among categories of Ni compounds suggest that health assessments for each of these categories are needed, including those in nano-size.

We recently assessed the genotoxicity of Ni and NiO NPs in comparison to soluble Ni by using a range of assays including comet assay, micronucleus assay, analysis of chromosome aberration and a set of reporter cells [6,7]. These studies showed that Ni and NiO NPs caused more pronounced DNA strand breaks [6], but all Ni exposures caused chromosomal damage via mechanisms that appeared to be calcium-dependent [7]. Except for genotoxicity, epigenetic alterations and gene expression changes are also proposed to be relevant mechanisms underlying the carcinogenicity of Ni exposure, as well as many other metal compounds [8]. Previous studies on various Ni compounds/particles have, e.g., shown the induction of signaling pathways involving PI3K (phosphatidylinositol 3-kinase) and Akt (Protein Kinase B), as well as hypoxia-inducible transcription factor-1 (HIF-1) [9,10,11]. Furthermore, gene expression changes following exposure of rats to NiSO_4_ or insoluble Ni have been investigated [12,13] but studies of wider transcriptional changes following exposure to Ni and NiO NPs are rare. Using a PCR array, Saquib and colleagues recently showed gene expression changes after HepG2 exposure to NiO NPs after acute (24 h) exposure to a relatively high dose (100 µg/mL) [14]. No study yet has revealed genome-wide changes following weeks of exposure to a relative low dose, an in vitro set-up that should be more relevant for human exposure. Furthermore, the role of cellular uptake and the relevance of the “Ni bioavailability model” has not been fully explored in studies focusing on gene expression changes.

**Figure 1:**
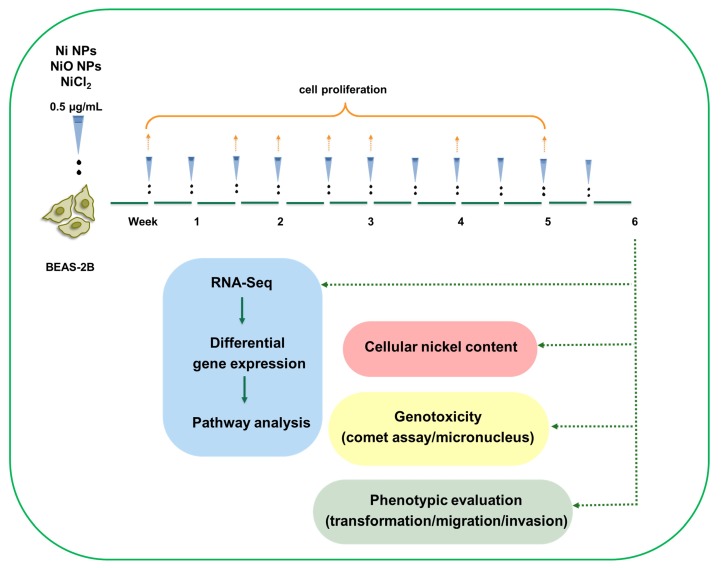
Low-dose, long-term exposure to nickel containing nanoparticle: experimental design. BEAS-2B cells were exposed to low doses (0.5 µg/mL) of Ni NPs, NiO NPs and NiCl_2_ as ion control over 6 weeks, with cells being split and cells re-exposed twice a week. During the six-week exposure, cell proliferation was assessed on a weekly basis. At the end of the six-week exposure, RNA sequencing was performed together with phenotypic evaluation (cell transformation, cell migration and cell invasion), genotoxicity (comet assay/micronucleus test) and cellular nickel content.

Thus, the aim of this study was to explore changes in gene expression using RNA sequencing following long term (six weeks) exposure of human lung cells to Ni and NiO NPs, as well as soluble NiCl_2_. Cell dose and endpoints of importance for carcinogenicity (i.e., DNA damage, soft agar colony formation, cell invasion and cell migration) were also evaluated. The overall experimental design is shown in Figure 1.

## 2. Materials and Methods

### 2.1. Nanoparticles and Characteristics

Nickel NPs (Ni, <100 nm), nickel(II) oxide NPs (NiO, <50 nm) and water soluble nickel(II) chloride (NiCl_2_·6H_2_O) were purchased from Sigma-Aldrich (St. Louis, MO, USA). The size and morphology of the same batch of Ni and NiO NPs have previously been reported [7] using transmission electron microscopy (TEM) showing Ni NPs to be predominantly less than 100 nm with a BET-area of 6.41 m^2^/g and the NiO NPs less than 50 nm and a BET-area of 102 m^2^/g. Particle dispersions were freshly prepared in cell medium (1 mg/mL) followed by 15 min sonication in an ultrasonic bath (Branson 2200). Subsequent dilutions were immediately prepared in serum-free cell medium prior to exposure. Both Ni and NiO NPs agglomerated in the cell medium, with an approximate average agglomerate size of 500 nm for the Ni NPs and 750 nm for NiO NPs [7]. Following incubation in cell medium at 37 °C (1 μg Ni/mL) for 48 h, similar amount of Ni was observed in solution for the two NPs, approximately 6% (normalized to the total amount of added Ni).

### 2.2. Cell Culture and Exposures

The immortalized human bronchial epithelial cell line (BEAS-2B, European Collection of Cell Cultures) was cultured in supplemented bronchial epithelial cell growth medium (BEGM, Lonza, Basel, Switzerland) on pre-coated flasks (0.01 mg/mL fibronectin, 0.03 mg/mL bovine collagen type I, 0.01 mg/mL bovine serum albumin and 0.2% penicillin-streptomycin in BEGM additive free medium). It should be noted that BEGM is serum-free and specially designed to support the growth of bronchial epithelial cells. BEAS-2B cells were independently authenticated according to ISO 9001-2004 and Mycoplasma tested (Leibniz Institute DSMZ, Braunschweig, Germany). BEAS-2B cells were seeded in six-well plates at an approximate density of 5 × 10^3^ cells/cm^2^. After 2 h, particle dispersions were directly added to the cell cultures to obtain a final mass concentration of 0.5 µg/mL based on mass of nickel (corresponding to 0.1 µg Ni/cm^2^). The final volume used in 6-well plates was 2 mL. For the six-week exposure, BEAS-2B were seeded in six-well plates (5 × 10^3^ cells/cm^2^, 2 mL cell medium per well) and allowed to attach for approx. 2 h. Thereafter, cells were exposed to Ni, NiO or NiCl_2_ in triplicates. Cells were split, counted, reseeded twice a week (5 × 10^3^ cells/cm^2^, 2 mL cell medium per well) and re-exposed.

### 2.3. Alamar Blue

Every week during the six-week exposure, cells were harvested, counted, seeded in 48-well plates (5 × 10^3^ cells/cm^2^), considering three technical replicates per each triplicate exposure. Cells were allowed to proliferate without exposure for 48 h. Medium was then removed, replaced with 10% Alamar Blue solution (ThermoFischer Scientific, Waltham, MA, USA) prepared in cell medium and allowed to incubate for 2 h at 37 °C. Fluorescence (Ex560/Em590) was recorded using a plate reader (Tecan Infinite F200) equipped with Magellan software. Background fluorescence (10% Alamar Blue in cell medium) was extracted from the total fluorescence of each well. Cell proliferation was calculated as % proliferation when the control was set to 100% and presented as mean values ± S.D. (*n* = 3).

### 2.4. Inductively Coupled Plasma Mass Spectrometry (ICP-MS)

Cellular uptake of Ni, NiO NPs and NiCl_2_ was quantified following three and six weeks of repeated exposures as previously described [7]. Briefly, BEAS-2B treated and untreated cells were washed with PBS, harvested, re-suspended in cell culture medium and counted. Samples were digested for 48 h in 32% HNO_3_ and were thereafter diluted to a final concentration of 2% HNO_3_ prior to the analysis. ^58^Ni and ^60^Ni isotopes were quantified using an iCAP Q ICP-MS (Thermo Scientific, Waltham, MA, USA). Nickel content was normalized according to the cell number and expressed as pg Ni/cell.

### 2.5. Comet Assay

The induction of DNA strand breaks and alkali labile sites following six weeks of exposure to 0.5 µg/mL of Ni, NiO NPs or NiCl_2_ was analyzed by the alkaline version of Comet assay as previously reported [7]. Results are presented as mean % of DNA in tail ± S.D.

### 2.6. Micronucleus Flow Cytometric Assay

Following six-week exposure of BEAS-2B to 0.5 μg/mL Ni NPs, NiO NPs, NiCl_2_ and 24 h exposure to 0.05 μg/mL mitomycin C (used as positive control) cells were seeded in 96-well plates and allowed to attach for approx. 2 h. Afterward the presence of micronuclei, hypodiploid nuclei induction as a marker of aneuploidy, cytotoxicity and cell cycle modulation were evaluated as previously described [15]. In vitro Microflow Kit (Litron Laboratories, Rochester, NY, USA) was used following manufacturer’s instruction and a BD Accuri C6 flow cytometer with flow set to 30 μL/min and 5000 gated nuclei events per triplicate samples were used for the analysis.

### 2.7. Invasion-Migration Assay

At the end of the six-week exposure of BEAS-2B to 0.5 µg/mL Ni, NiO NPs or NiCl_2_, cells were harvested, counted and seeded in BEGM medium without supplements in BioCoat Matrigel invasion chambers, as well as in uncoated transwell inserts (8 µm pore size, polycarbonate, 0.33 cm^2^ insert surface area). BEGM medium with supplements was added to the basolateral compartments to act as a chemoattractant. After 48 h, incubation cells were washed and fixed in 4% formaldehyde for 15 min and stained with a 10% Giemsa solution for 20 min. Cells on the tops of insert membranes were removed by wiping with cotton swabs and five pictures were taken for each insert using a Nikon ECLIPSE TE2000-S microscope (10× magnification). Images were scored by using NIH Image J software and the number of migratory and invading cells were counted. TGF-β (15 ng/mL 72 h exposure) was used as positive control.

### 2.8. Soft Agar Cell Transformation and Colony-Forming Efficiency Assays

Anchorage independent cell growth and the clonogenicity of treated and untreated BEAS-2B were assessed following six-week exposures in parallel experiments, as previously described [16]. Results are expressed as the mean number of colonies per treatment for both assays as well as the transformation frequency (%) to account for the plating efficiency obtained in colony-forming efficiency assays when calculating the transformation index.

### 2.9. RNA Extraction

At the end of the six-week exposure, total RNA was extracted using the RNeasy Mini Columns (Qiagen, Hilden, Germany) in accordance with manufacturer’s instructions (including the optional purification step with DNase I). Total RNA concentration was determined spectrophotometrically using NanoDrop (NanoDrop Technologies, Wilmington, DE, USA). The quality control of the RNA samples was conducted using the Bioanalyzer 2100 (Agilent Technologies, Santa Clara, CA, USA) and all samples had RIN values above eight.

### 2.10. RNA Sequencing and Data Analysis

RNA sequence libraries were generated with standard mRNA stranded protocols from Illumina and sequenced on a Hiseq2500 (pair end reads 101 bp long, RapidRun mode) at the Science for Life Laboratory, Stockholm, Sweden. Data processing was carried out at SNIC-UPPMAX, Uppsala, Sweden [17]. The generated reads were mapped to the human genome version GRCh37 using Tophat v. 2.0.4 [18]. Read data were converted to gene counts with the program htseq v. 0.6.1 [19]. Differential gene expression was assessed using Bioconductor DESeq2 package [20] running in R statistical programme language version 3.2.0. Only genes with *p*-values lower than 0.05 after correction for multiple testing (false discovery rate, FDR) were treated as differentially expressed. The sequencing data were deposited at ArrayExpress, (E-MTAB-8903). Venn diagrams of the differentially expressed genes for each treatment versus the untreated control were plotted with a web tool developed by the Bioinformatics & Evolutionary Genomics Laboratory at VIB/UGent, Belgium (http:bioinformatics.psb.ugent.be/webtools/Venn/). The lists of genes for plotting the Venn diagrams were based on the Ensembl gene ID.

### 2.11. Pathway, Network and Gene Enrichment Analysis

Ingenuity Pathway Analysis (IPA) software (license obtained from Ingenuity Systems, Redwood City, CA, USA) was used to perform canonical pathway analysis. Analysis was performed on all the differentially expressed genes (FDR-adjusted *p*-value < 0.05). Heatmaps were generated using data output from IPA pathway analysis with genes ordered according to their average fold change.

### 2.12. Statistical Analysis

Differences between groups were evaluated by one-way ANOVA followed by Dunnet’s post-hoc test (for comparisons with the control) or by t-test (for comparisons between two groups). For the cell cycle analysis, differences between groups were evaluated by two-way ANOVA followed by a Bonferroni post-hoc test. All analyses were performed using GraphPad Prism version 5.02. *p* <  0.05 was considered statistically significant.

## 3. Results

### 3.1. Ni and NiO NPs, but Not Soluble Ni, Are Readily Taken up by Human Lung Cells

**Figure 2:**
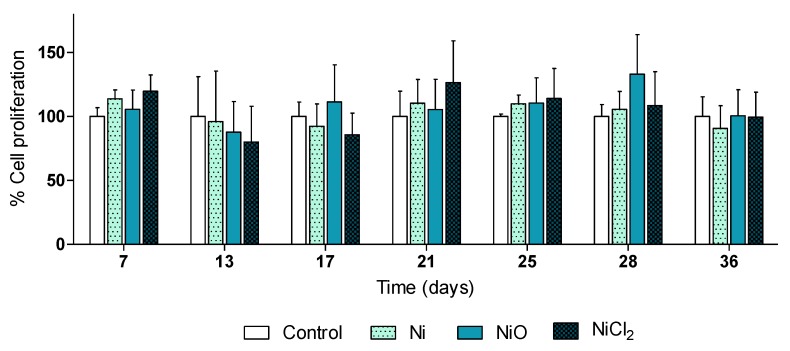
Human lung cells (BEAS-2B) were exposed to Ni NPs, NiO NPs and NiCl_2_ (0.5 µg/mL) for 6 weeks. At the indicated time-points Alamar Blue proliferation assay was performed. Results are presented as mean values ± S.D.

In this study, the aim was to analyze effects at relatively low doses without overt cytotoxicity. In line with this, results on cell viability showed that repeated exposure to 0.5 µg/mL Ni and NiO NPs, or the corresponding soluble Ni salt (in form of NiCl_2_), for 6 weeks did not affect cell viability/proliferation of BEAS-2B cells at any of the investigated timepoints (Figure 2).

**Figure 3:**
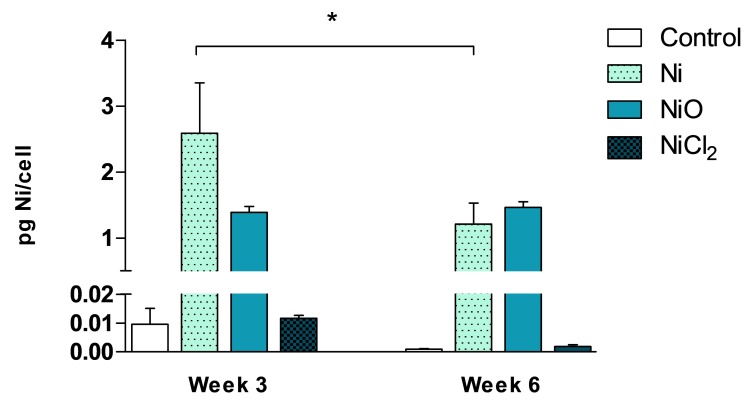
Cellular nickel content was quantified by ICP-MS after 3 and 6 weeks of exposure. Results and expressed as pg Ni/cell and are presented as mean values ± S.D. (*n* = 3). Significant results are indicated with asterisks (* *p*-value < 0.05).

We used ICP-MS to evaluate Ni cellular content at an intermediate time-point (3 weeks) and at the end of the exposure (6 weeks). The results showed similar Ni content per cell (1.5–2.5 pg Ni/cell) after exposure to Ni and NiO NPs and this did not increase at week six compared to week three (Figure 3). Instead, slightly lower levels were observed for Ni NPs. In contrast to the NPs, no increase in cellular Ni compared to controls were observed after exposure to NiCl_2_.

### 3.2. RNA Sequencing Reveals Gene Expression Changes Following Six-Week Exposure of BEAS-2B Cells to Ni

**Figure 4:**
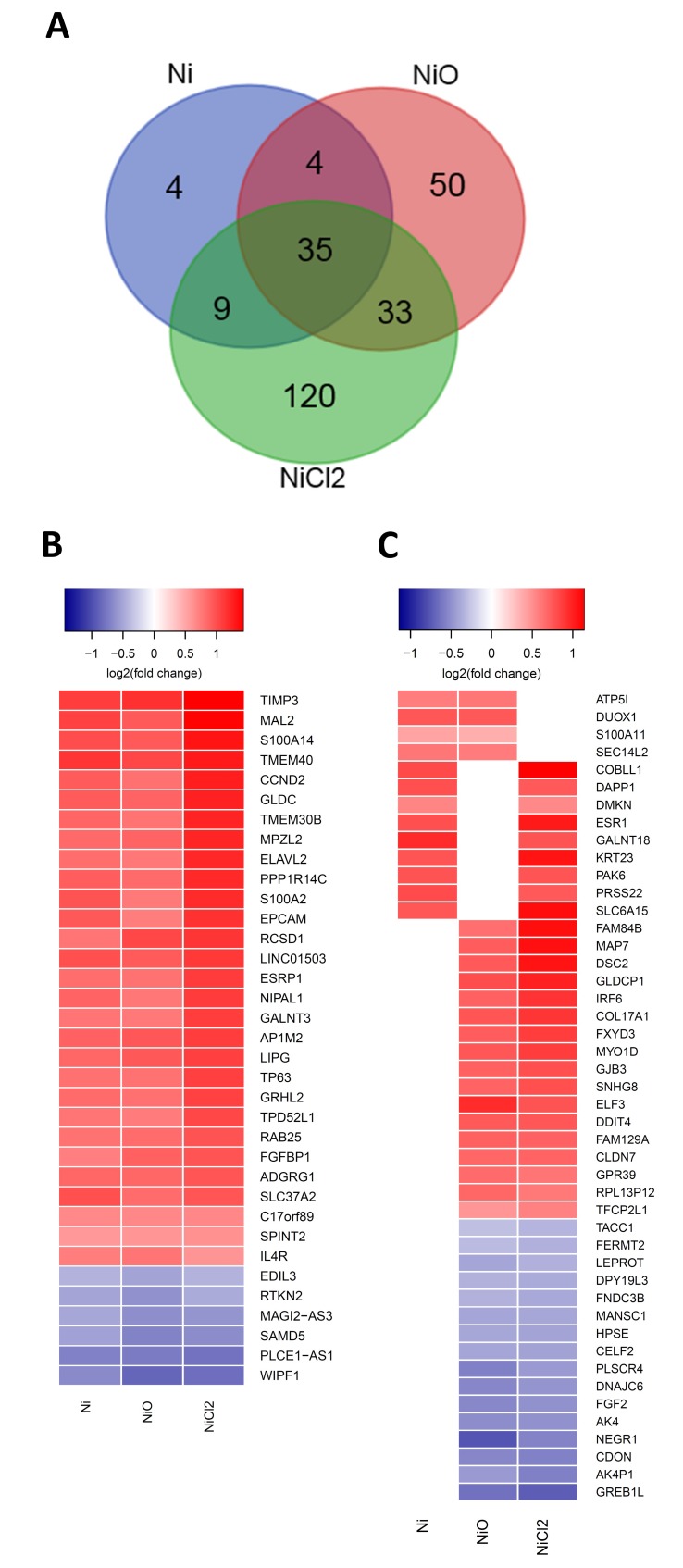
RNA sequencing analysis of the BEAS-2B cells exposed to Ni NPs, NiO NPs or NiCl_2_. (**A**) Venn diagrams of the differentially expressed genes (genes that have a false discovery rate (FDR)-adjusted *p*-value < 0.05 are considered differentially expressed). (**B**) Heatmap of the differentially expressed genes that overlap between the Ni, NiO and NiCl_2_ exposures. (**C**) Combined heatmap of the differentially expressed genes shared between two of the exposures. Color indicates log2(fold change).

Changes in gene expression in BEAS-2B cells were evaluated by RNA sequencing at the end of the six-week exposure. NiCl_2_ treatments resulted in the largest number of differentially expressed genes (DEGs, false discovery rate adjusted *p*-value < 0.05), namely 197 DEGs (144 upregulated, 53 downregulated), followed by NiO nanoparticles with 122 DEGs (74 upregulated, 48 downregulated) and Ni nanoparticles 52 DEGs (44 upregulated, eight downregulated). There were 35 DEGs that overlapped between all the treatments and four genes that overlapped only between the nanoparticle treatments (and not NiCl_2_) (Figure 4A). Notably the direction of the gene expression changes was similar between the treatments that overlapped (Figure 4B,C). One of the most differentially expressed genes, *TIMP3*, coding for the tissue inhibitor of metalloproteinase-3, a glycoprotein with tumor-suppressing properties [21] was upregulated by all treatments. Genes coding for calcium-binding proteins such as *S100A14* and *S100A2* were also upregulated by all treatments, whereas *S100A11* was upregulated by Ni and NiO nanoparticles only. Calcium-binding proteins are often dysregulated in lung carcinomas [22]. Gene expression of cyclin 2 *CCND2*, which is involved in the regulation of cell cycle and oncogenic transformation [23], as well as epithelial cell adhesion molecule *EPCAM*, that is altered in epithelial tumors [24], were also upregulated by all treatments. In addition, *TP63*, a transcription factor involved in regulation of epithelial morphogenesis [25] and interleukin-4 receptor *IL4R*, a receptor overexpressed in several epithelial cancers [26] had their gene expression upregulated by all treatments. Expression of DNA Damage Inducible Transcript 4 (*DDIT4*), was upregulated by NiO NPs and NiCl_2_.

### 3.3. Pathway Enrichment and Upstream Regulator Analysis

**Figure 5:**
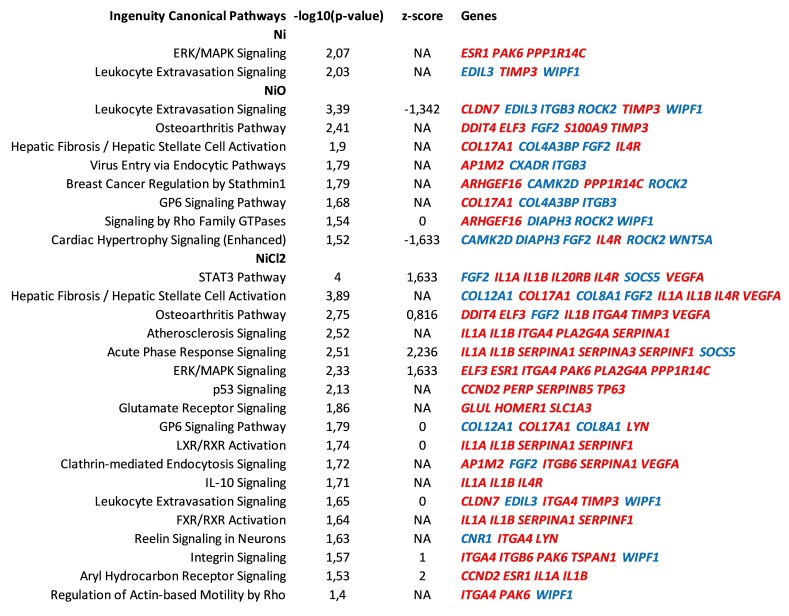
Canonical pathways enriched after six-week exposure of BEAS-2B cells to Ni, NiO nanoparticles or NiCl_2_. Pathway analysis was performed in IPA on the differentially expressed genes following exposure of BEAS-2B cells to Ni, NiO nanoparticles or NiCl_2_. Significantly enriched canonical pathways (−log10 (*p*-value) > 1.3) are illustrated, ordered according to the statistical significance. Pathways containing less than three differentially expressed genes were excluded. Some pathways are additionally characterized by z-score, a measure of the activation state of the pathway. NA, z-score not available; color coding indicates direction of differential expression, red—upregulation, blue—downregulation.

Canonical pathway analysis, as well as upstream regulator analysis, of the differentially expressed genes was performed using Ingenuity Pathway Analysis software. While extracellular signal–regulated kinase (ERK)/MAPK signaling and Leukocyte Extravasation signaling were the only two pathways enriched for Ni NPs, one should keep in mind that the number of differentially expressed genes was relatively low, i.e., 52 for a robust downstream analysis (Figure 5). Leukocyte extravasation signaling was the top enriched pathway for NiO NPs and was denoted by genes such as *TIMP3* (upregulated) and integrin-B3 (*ITGB3*, downregulated) (Figure 5). An enriched pathway for NiO NPs was (Hepatic) Fibrosis that was defined by, e.g., collagen-17A1 (upregulated) and collagen-4A3 binding protein (downregulated) (Figure 5). STAT3 pathway, the top enriched pathway for NiCl_2_, was defined by, e.g., interleukin-1A and -1B (*IL1A*, *IL1B*, upregulated) and Vascular Endothelial Growth Factor A (*VEGFA*, upregulated). The acute phase response signaling pathway was enriched after NiCl_2_ with a high positive z-score (2.2) that is predictive of pathway activation and was mostly defined by upregulated genes such as interleukin-1A and -1B (*IL1A*, *IL1B*), as well as genes belonging to the serpin family of protease inhibitors (*SERPINA1*, *SERPINA3*, *SERPINF1*).

Upstream regulator analysis indicated that ZEB1, SNAI1 and TP63 are transcription factors that are predicted upstream regulators for all treatments (Supplementary Table S1). ZEB1 and SNAI1 had a negative score, indicating that their inhibition is predicted to explain part of the gene expression changes. On the other hand, TP63 had a positive score, indicating that its activation is predicted to explain some of the gene expression changes (Figure 5). In addition, *TP63* was differentially expressed (upregulated) by all treatments (Figure 4B). A network was generated around the aforementioned transcription factors for NiCl_2_, as the number of DEGs was highest for this treatment (Supplementary Figure S1). Regarding the upstream regulators as cytokines, TNF, IL1B and IL4 were all predicted as being activated (positive z-score) for NiCl_2_ and, in addition, IL4 was also predicted for Ni NPs.

### 3.4. Nickel-Containing NPs Induce DNA Strand Breaks and Alter Cell Cycle after Six Weeks of Repeated Exposure in BEAS-2B Cells

**Figure 6:**
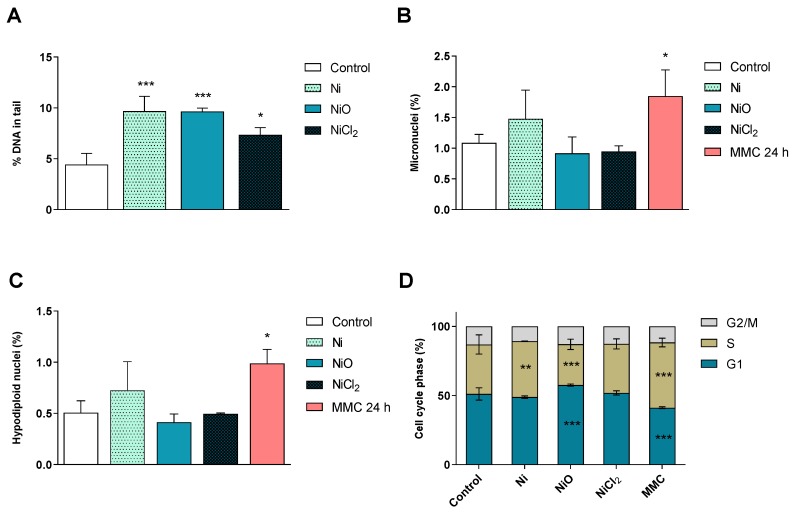
Genotoxicity and cell cycle analysis. (**A**) Induction of DNA strand breaks after six-week exposure was investigated using the alkaline comet assay. Results are expressed as % DNA in tail and presented as mean ± S.D. (*n* = 3). (**B**–**C**) Micronuclei formation after six-week exposure was evaluated by flow cytometry. Results are expressed as percentage of micronuclei (**B**) and hypodiploid nuclei (**C**) and presented as mean values ± S.D. (*n* = 3). (**D**) Cell cycle phase evaluation was performed by flow cytometry after 6 weeks of exposure. Results are presented as mean distribution (%) of the different cell cycle phases (G1, S, G2/M) ± S.D. (*n* = 3). Positive controls: MMC 24 h, mytomycin C, 0.05 µg/mL 24 h. Significant results are marked with asterisks (* *p*-value < 0.05, ** *p*-value < 0.01, *** *p*-value < 0.001).

At the end of the six-week repeated Ni exposures, DNA damage was evaluated by using the comet assay and a flow-cytometry version of the micronucleus assay. The results showed that all treatments induced DNA strand breaks (Figure 6A). The DNA damage was, however, not translated into chromosomal breakage (micronuclei, Figure 6B) or loss (hypodiploid nuclei, Figure 6C). Cell cycle phases were also evaluated in the same extensive flow-cytometry assay. Ni NPs increased the number of cells in the S phase, while NiO NPs increased the number of cells in the G1 phase, as well as decreased the S-phase compared to the control (Figure 6D). However, none of the treatments induced apoptosis or necrosis (data not shown).

### 3.5. No Clear Changes in Cell Transformation or Cell Motility

**Figure 7:**
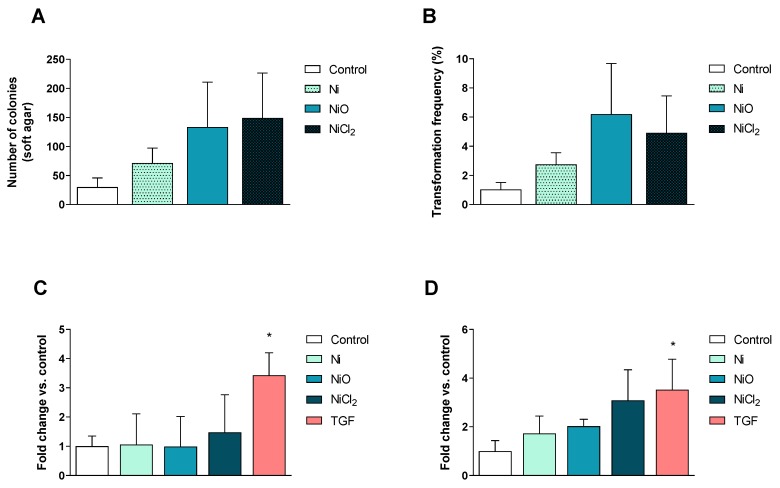
Cell transformation and motility. (**A**–**B**) Soft agar cell transformation was determined after 6 weeks of exposure. Results are expressed as total number of colonies (**A**) as well as the overall transformation frequency (**B**) in which the colony-forming efficiency was considered (mean ± S.D., *n* = 3). Cell migration (**C**) and invasion (**D**) was evaluated after 6 weeks of exposure and results are expressed as fold change compared to the control presented as mean ± S.D. (*n* = 3). Positive control: TGFβ, 15 ng/mL 72 h. Significant results are marked with asterisks (* *p*-value < 0.05).

At the end of the six-week exposure, the ability of the different Ni exposures to affect cell transformation (ability to form colonies in soft agar), cell migration, as well as cell invasion, was evaluated. There were more colonies formed in soft agar for all Ni exposures, but the variation was large, leading to no statistically significant changes (Figure 7A). Concurrently, we also evaluated the colony-forming efficiency of the cells. Only NiO NPs reduced the colony-forming efficiency (data not shown) but the transformation index (which accounts for the plating efficiency and clonogenicity observed in the colony-forming efficiency assay) was not altered for any of the treatments (Figure 7B). No changes in cell migration were observed (Figure 7C). A slight increase in cell invasion was noted but the variation was high and statistical significance was not reached (Figure 7D).

## 4. Discussion

Even though the toxicity of Ni and NiO NPs has been studied in various cell models, no study has, thus far, revealed cellular effects, including transcriptomic profiling, following weeks of exposure to a relatively low dose of such NPs with a comparison of effects to soluble Ni. One of the most important findings in this study is the clear effect on transcriptome for NiCl_2_, despite the limited uptake. This suggests that the transcriptional changes are mediated via cell membrane receptors, a mechanism that we recently suggested to be of importance also for micronucleus formation [7]. In our previous study, we provided evidence of a mechanism that included interference with Ca^2+^ signaling. The transcriptomic data supports the involvement of such mechanisms since genes coding for calcium-binding proteins, such as *S100A14* and *S100A2*, were upregulated by all Ni exposures.

The role of extracellular- and calcium-dependent mechanisms has also been proposed in other reports on toxicity of Ni. For example, a study using THP-1 cells to investigate mechanisms for Ni-induced changes on IL-8 gene expression demonstrated the role of an extracellular mechanism via TLR-4 and the L-type Ca^2+^ channel, followed by the downstream extracellular signal-regulated kinases (ERKs) and NF-κB signaling pathways [27]. Indeed, ERK/MAPK signaling was predicted in the IPA analysis for Ni NPs and NiCl_2_ in our study (Figure 5). Previous studies have also suggested a role for PI3K/Akt and ERK signaling followed by Vascular Endothelial Growth Factor (VEGF) expression as a response to Ni [9,28,29]. We also recently reported the secretion of VEGF in THP-1-derived macrophages following exposure to Ni and NiO NPs [30] and, in the present study on BEAS-2B cells, increased transcription of *VEGFA* was observed for NiCl_2_ (see Figure 5). Taken together, this and previous studies suggest the importance of extracellular receptors and calcium signaling for Ni-induced gene expression changes.

Except for calcium-binding proteins, *TIMP-3* (coding for an enzyme involved in inhibiting metalloproteinases) was one of the most differentially expressed genes in all three Ni exposures, indicating a general role of metalloproteinases (MMPs) in Ni-induced toxicity. MMPs cleave components of the extracellular matrix as well as other substrates, including cytokines and cell surface adhesion receptors [31]. Previous studies have shown increased expression of MMPs in bronchoalveolar lavage following exposure of mice to Ni NPs [32], in human monocytes exposed to Ni NPs [33] as well as in the nasal lavage fluid of welders exposed to nickel-containing welding particles [34]. The two latter studies also found increased expression of TIMP-1. In a study on Ni-refinery workers, IL1A, a cytokine known to be elevated in many tumor types, was the most upregulated gene in the blood cells [35]. Increased expression of this gene was observed in our study for NiCl_2_, as were other genes involved in inflammation such as *IL1B* and *SERPINA* (canonical pathways related to atherosclerosis and acute phase signaling).

In terms of animal experiments, gene expression changes in the lungs of rats exposed to NiSO_4_ showed, for example, upregulation of the inflammatory gene *Ptgs2* (COX-2) and Interleukin receptor 7 (*Il7r*) as well increased expression of genes related to oxidative stress (*Nfe2l2*, *Hmox1*) and proliferation (cyclins) [13]. Similarly, increased expression of cyclin 2 (*CCND2*), as well as and interleukin-4 receptor *IL4R*, was observed for all Ni-treatments in our study. The transcription factor p63, important for epithelial differentiation [36], was also increased for all Ni exposures in our study. In line with this, a transcriptional target to p63, *DDIT* (also known as REDD1) [36] was increased for NiO and NiCl_2_. DDIT has been shown to be induced in response to DNA damage and hypoxia and has, furthermore, been shown to cause an inhibition of mTOR, a kinase involved in PI3K/Akt signaling [37]. Increased expression of *DDIT* has previously been observed for NiO NPs in HepG2 cells [14].

Except for the gene expression changes observed, the present study also showed that even this low dose caused an increase in DNA breaks measured using the comet assay, similar to what was previously observed for higher doses of Ni exposure in a short-term study on BEAS-2B [7]. In contrast, we did not observe any micronucleus induction in this study. It is possible that low levels may be harder to detect using the flow cytometry version used in this study, although our previous study on various TiO_2_ NPs showed good consistency between scoring using microscopy and flow cytometry [15]. In the present study, low dose and long-term Ni exposures did not show clear phenotypical changes in terms of cell migration, invasion and transformation ability. This is in contrast with previous studies using higher doses showing, e.g., that NiCl_2_ promotes the invasive potential of lung cancer cells via the activation of TLR4 signaling following acute exposures [38]. Furthermore, in order to transform BEAS-2B cells, exposure to 100 µM NiCl_2_ up to 12 months were needed in previous studies [39,40]. Additional studies are needed to elucidate the role of cell type, dose and exposure time on molecular mechanisms for nickel-induced cell transformation.

Taken together, by using RNA sequencing, this study shows clear gene expression changes following the long-term (six-week) exposure of human lung cells to Ni and NiO NPs, as well as soluble NiCl_2_. Importantly, the most pronounced changes were observed for NiCl_2_, despite the lack of or limited uptake, suggesting the major role of extracellular receptors and downstream signaling. Genes changed for all Ni exposure included genes coding for calcium-binding proteins (*S100A14* and *S100A2*) and genes including *TIMP3*, *CCND2*, *EPCAM*, *IL4R* and *DDIT4*. To some extent, these results challenge the Ni bioavailability model, since transcriptional changes take place in the absence of Ni in the nucleus. Nevertheless, it is important to note that the toxicological effects of the NPs can still be more severe due to more pronounced lung retention.

## Supplementary Materials

The following is available online at https://www.mdpi.com/2079-4991/10/4/649/s1, Supplementary Table S1: Upstream regulator analysis; Supplementary Figure S1: Network around top upstream regulators for NiCl_2_.
