# Supplementary material for: Transcriptome Profiling and Toxicity Following Long-Term, Low Dose Exposure of Human Lung Cells to Ni and NiO Nanoparticles—Comparison with NiCl2
Source: Nanomaterials (Basel). 2020 Mar 31;10(4):649. doi: 10.3390/nano10040649 (PMC7221965; doi:10.3390/nano10040649)
Supplement: Supplementary file 1 [file nanomaterials-10-00649-s001.pdf]

Supplementary Table 1. Upstream regulator analysis

Upstream Regulator	Expr Log Ratio	Molecule Type	Activation z-score	p-value of overlap	Target Molecules in Dataset
**Ni**					
ZEB1		TR	-2.1	2.5E-09	ESRP1,GRHL2,MAL2,MPZL2,RAB25,TP63
SNAI1		TR	-2.0	1.5E-05	CCND2,ESRP1,RAB25,TP63,WIPF1
CTNNB1		TR	2.0	2.6E-04	CCND2,EDIL3,EPCAM,FGFBP1,GRHL2,MPZL2,TIMP3,TP63
TP63	0.8	TR	1.9	2.9E-04	CCND2,GRHL2,MPZL2,S100A2,TIMP3,TP63
KLF4		TR	2.0	2.3E-03	CCND2,COBLL1,EPCAM,S100A14
IL4		C	2.1	4.9E-03	CCND2,COBLL1,DUOX1,IL4R,LIPG,SPINT2,TIMP3
**NiO**					
ZEB1		TR	-1.5	3.3E-10	CXADR,ESRP1,GRHL2,MAL2,MPZL2,RAB25,ST14,TP63
SNAI1		TR	-1.7	6.7E-06	CCND2,CLDN7,CXADR,ESRP1,RAB25,TP63,WIPF1
SOX2		TR	1.5	1.5E-05	CLDN7,ELF3,EPCAM,IRF6,RNF43,SAPCD2,TEAD1,TIMP3,TP63,WIPF1,WNT5A
TP63	0.8	TR	2.3	1.1E-03	CCND2,DDIT4,GRHL2,MPZL2,S100A2,TIMP3,TP63,WNT5A
**NiCl2**					
TP63	1.1	TR	3.6	7.4E-11	CCND2,CDH3,CSTA,DDIT4,GRHL2,HAS3,IL1B,ITGA4,LYN,MIR205HG,MPZL2, PARD6G,PERP,S100A2,SERPINB5,SERPINF1,SRD5A1,TIMP3,TP63,VEGFA,ZEB2
TP73		TR	1.9	5.1E-08	CCN5,DDIT4,FGF2,HPSE,IGSF3,IL1B,IL4R,MPZL2,PERP,S100A2,SERPINA1, SERPINA3,SERPINF1,THBS2,TIMP3,TSPAN1,VEGFA
TNF		C	2.9	2.1E-07	BIRC3,CCN5,CCND2,CDH3,CLDN7,DSC2,DSC3,ELF3,EPCAM,ESR1,FAT2,FERMT1, FGF2,HAS3,IL1A,IL1B,IL4R,ITGA4,ITGB6,KRT23,LYN,MFSD2A,MPC1,OCLN, PLA2G4A,PRRG4,RAPGEF5,SAMD4A,SERPINA3,SERPINF1,SFRP1,SLC1A3,SNCG, THBS2,TIMP3,TMEM40,TP63,VEGFA
ZEB1		TR	-2.3	4.8E-07	ESRP1,GRHL2,MAL2,MPZL2,RAB25,RBM47,TP63
TP53		TR	2.1	5.2E-07	AK4,BIRC3,CCN5,CCND2,CDH3,CLCA2,DDIT4,DSC3,EDIL3,ESR1,FAT2,FERMT2, FGF2,FGFBP1,FXYD3,GJB3,GLUL,GPR87,H2AFY2,IL1A,IL1B,IL4R,MPZL2,PARD6G, PERP,PSTPIP2,S100A2,SERPINA3,SERPINB5,SFRP1,SRGAP3,THBS2,TIMP3,TP63, TPD52L1,VEGFA,ZEB2
SNAI1		TR	-2.0	3.6E-06	CCND2,CLCA2,CLDN7,ESRP1,OCLN,RAB25,TP63,WIPF1,ZEB2
EZH2		TR	2.1	1.0E-05	BIRC3,CCND2,CNR1,FHOD3,HOXA1,MAL2,MPZL2,PLA2G4A,RASSF9,SERPINA1, SLC1A3,TIMP3,VEGFA
PPP1R13L		TR	2.0	1.8E-05	CCND2,DSC3,PERP,TP63
GLI1		TR	1.6	5.1E-05	CCND2,ELAVL2,EMB,HPSE,PSTPIP2,SESN3,SFRP1,TIMP3,VEGFA
FOS		TR	1.5	5.8E-05	CDON,CELF2,COBLL1,CSTA,CTSV,DSG3,EMB,FGF2,HOOK1,HOXA1,IL1A,MPZL2, PLA2G4A,SNCG,VEGFA
BRCA1		TR	2.4	1.2E-04	CDH3,DDIT4,ESR1,FXYD3,PERP,S100A2,TP63,VEGFA
JUN		TR	1.9	1.5E-04	BIRC3,CCND2,CSTA,FGF2,HOMER1,IL1A,IL1B,PLA2G4A,SERPINB5,SNCG,TIMP3, VEGFA,ZEB2
SREBF1		TR	1.9	3.0E-04	ADGRG1,AK4,DPY19L3,IL1A,IL1B,SERPINA1,SERPINA3,SLC22A5,VEGFA
KLF4		TR	1.7	3.5E-04	CCND2,CLDN7,COBLL1,COL8A1,EPCAM,IRF6,S100A14,SERPINA1,VEGFA
STAT3		TR	1.7	3.7E-04	CCND2,CDON,ESR1,FERMT2,FGF2,IL1B,IL4R,ITGB6,PLA2G4A,SERPINA1,SERPINA3, SERPINB3,SLC1A3,VEGFA
CBX5		TR	-2.4	4.0E-04	FGFBP1,MAL2,PROM2,SERPINA3,TACSTD2,TMEM30B
RELB		TR	-2.0	3.4E-03	BIRC3,ESR1,IL1B,SERPINB5
IL17A		C	1.8	4.5E-03	EDIL3,FGF2,IL1A,IL1B,OCLN,TP63,VEGFA
CSF1		C	2.2	1.2E-02	CCND2,IL1A,IL1B,ITGA4,SFRP1,VEGFA
IL1B	0.8	C	1.6	1.6E-02	BIRC3,DDIT4,ELF3,ESR1,FAM129A,FGF2,HOMER1,IL1A,IL1B,OCLN,PLA2G4A, SERPINA3,SLC1A3,TIMP3,VEGFA
RUNX1		TR	2.0	1.7E-02	ADGRG1,FAT4,IL1B,ITGA4,VEGFA
CREB1		TR	2.0	1.8E-02	AK4,CACNA2D1,EDIL3,HOMER1,IL1B,MFSD2A,PLA2G4A,SRD5A1,ST6GALNAC5,VEGFA
IL4		C	2.0	3.8E-02	CCND2,CELF2,COBLL1,IL1A,IL1B,IL4R,ITGA4,LIPG,RAB30,SAMD4A,SERPINB3,SPINT2, TIMP3,VEGFA
ATF4		TR	2.0	4.4E-02	DDIT4,SERPINF1,SLC3A2,VEGFA

Upstream regulator analysis was performed in IPA on the differentially expressed genes following 6 week exposure of BEAS-2B cells to Ni, NiO nanoparticles or NiCl2. Significantly predicted upstream regulators of the type transcriptional regulator and cytokine that had a predicted absolute activation z-score >1.5 are included. TR, transcriptional regulator; C, cytokine.

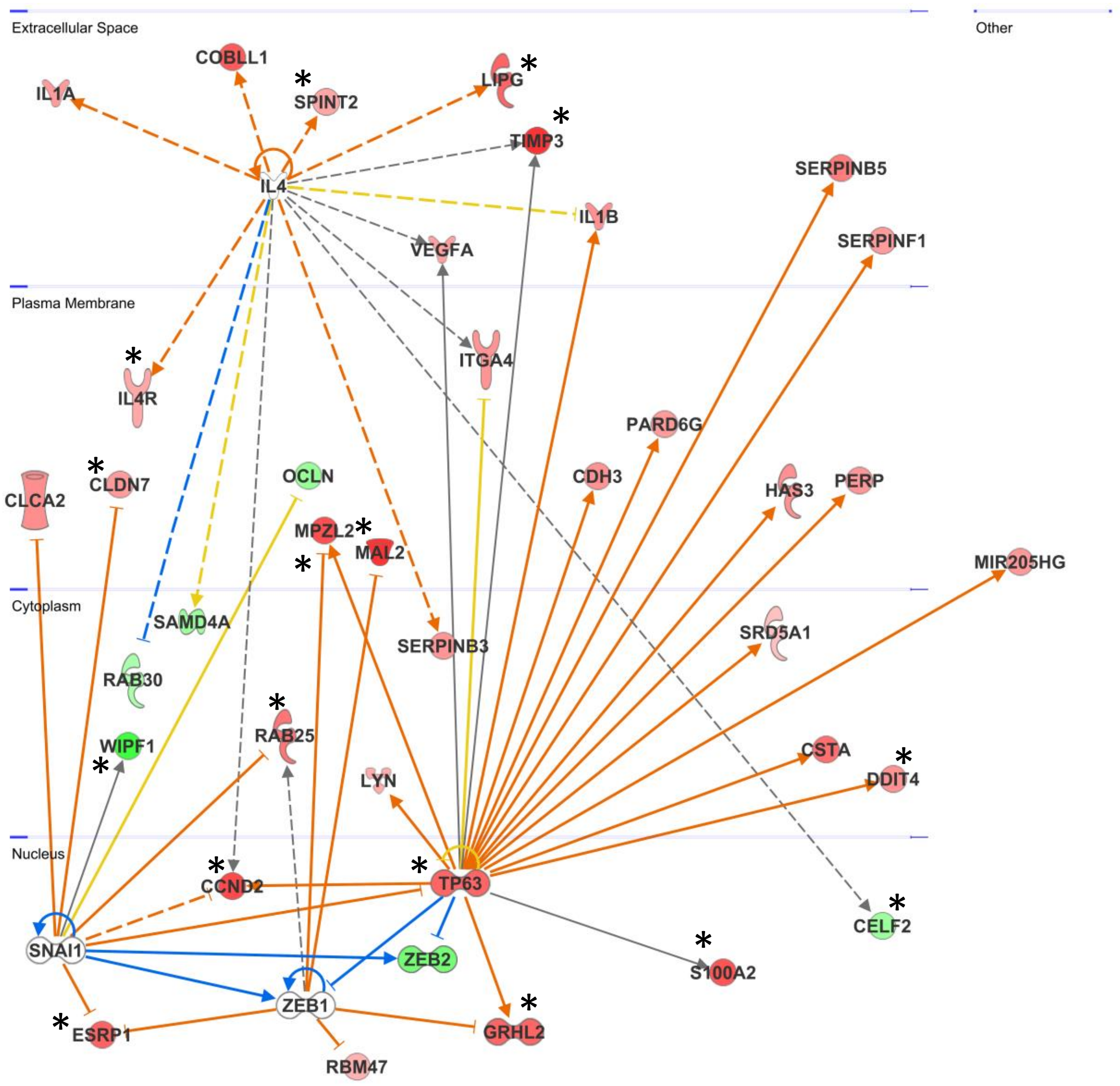

Supplementary Figure 1. Network around top upstream regulators for NiCl2. Upstream regulator analysis was performed using the IPA software tool and a network was generated for the predicted top transcription factors, ZEB1, TP63, SNAI1 and IL4. Asterisk indicates genes that are also differentially expressed after NiO treatment.
